# Sifter-T: A scalable framework for phylogenomic probabilistic protein domain functional annotation

**DOI:** 10.1186/1471-2105-16-S8-A4

**Published:** 2015-04-30

**Authors:** Danillo C Almeida-E-Silva, Ricardo Z N Vêncio

**Affiliations:** 1 Universidade de São Paulo, São Paulo, Brazil

## Background

In the functional annotation field, Sifter v2.0 is regarded as one of the best when it comes to annotation quality. Recently, it has been considered one of the best tools for functional annotation according to the initiative “Critical Assessment of Protein Function Annotation” (CAFA), an open collaborative experiment designed for large-scale assessment of protein function prediction tools. Sifter combines two powerful ideas: phylogenomics and bayesian graphical models. Nevertheless, it is still not widely used. This contradictory observation is probably due to issues with usability and suitability of the framework to a high throughput scale.

Although powerful in terms of approach, it can be considered prototype level in terms of software. The current Sifter version does not allow nucleotide or amino acid sequences input directly, nor accepts current standards in gene annotation formats. Moreover, several parameters are still hardcoded and difficult to be tuned by the end user. Finally, its relationship to third party dependence software is cumbersome, along with its output.

## Description

In this study, we had two goals: (i) enhance the tool’s usability, through local implementa- tions or a web-based front end; and (ii) optimize the original source-code for better performance, allowing it to be used in genome-wide scale.

Among the implemented strategies we have: parallel threads; CPU load balancing; best use of disk access, memory usage and runtime; adaptation to the currently used biological databases formats; improved user accessibility; expansion of accepted input types; automation of the reconciliation process; new output format; detailed documentation; and other minor implementations.

The increased performance allowed, for example, the reannotation of 419,029 Saccharum officinarum (sugarcane) ESTs to be performed by Sifter-T in 5 days, while BLAST took 49 days in a standard bioinformatics laboratory machine.

## Conclusions

This implementation result is presented as Sifter-T (Sifter Throughput-optimized), an open source tool with better usability and performance when compared to the original Sifter workflow implementation. The new Sifter-T features allow researchers to have easy and quick access to the Sifter’s powerful annotation mathematical method, now with enhanced experiment customization and keeping the inference engine intact. Sifter-T, and its online interface, is freely available at http://labpib.fmrp.usp.br/methods/sifter-t/.

